# Finite Element Method-Based Simulation Creep Behavior of Viscoelastic Carbon-Fiber Composite

**DOI:** 10.3390/polym13071017

**Published:** 2021-03-25

**Authors:** Mostafa Katouzian, Sorin Vlase, Maria Luminita Scutaru

**Affiliations:** 1 Department Machine Tools, Technical University of Munich, 85748 Munich, Germany; d-mec@unitbv.ro; 2 Department of Mechanical Engineering, Transilvania University of Brașov, B-dul Eroilor 20, 500036 Brașov, Romania; 3 Romanian Academy of Technical Sciences, B-dul Dacia 26, 030167 Bucharest, Romania

**Keywords:** finite element method, reinforced composite, viscoelasticity, creep response, engineering constants, carbon fiber, epoxy matrix

## Abstract

Usually, a polymer composite with a viscoelastic response matrix has a creep behavior. To predict this phenomenon, a good knowledge of the properties and mechanical constants of the material becomes important. Schapery’s equation represents a basic relation to study the nonlinear viscoelastic creep behavior of composite reinforced with carbon fiber (matrix made by polyethrtethrtketone (PEEK) and epoxy resin). The finite element method (FEM) is a classic, well known and powerful tool to determine the overall engineering constants. The method is applied to a fiber one-directional composite for two different applications: carbon fibers T800 reinforcing an epoxy matrix Fibredux 6376C and carbon fibers of the type IM6 reinforcing a thermoplastic material APC2. More cases have been considered. The experimental results provide a validation of the proposed method and a good agreement between theoretical and experimental results.

## 1. Introduction

Creep phenomena are often encountered in engineering and occur in viscoelastic materials, representing a permanent deformation that occurs due to a long mechanical stress. This phenomenon is generally time dependent. The deformation rate depends on several factors, first on the properties of the material and then on the temperature, the level of stress [1,2,3,4,5]. Temperature is one of the main factors that can significantly increase the deformation rate. The creep phenomenon can sometimes be undesirable. A significant increase usually occurs near the melting point, but there are also materials in which this phenomenon can manifest itself at room temperature. Polymers are one of these materials. The study of creep phenomena in composite materials has been intense in recent decades, especially due to considerations related to the great practical use of these materials in the automotive industry, civil or aeronautical but also in other fields. In the design phase, if it is known that this creep phenomenon can occur, it is necessary to know the deformation rate. In general, these data are obtained experimentally, but there are also theoretical models, which allow obtaining the behavior of a material under certain conditions of temperature and stress [6,7,8,9,10,11,12,13,14,15,16,17,18]. For a correct study of these creep phenomena, information is needed to refer to the mechanical properties of the studied materials. 

This information can be obtained from experimental measurements, but it is more economical if numerical calculation methods are used, experimentally validated. We present some results obtained taking into account this observation. To obtain the behavior of structures manufactured by of linearly non-aging viscoelastic and highly heterogeneous phases a numerical multiscale method is presented in [19]. The method chosen, uses the classic representative volume element (RVE) selected for the composite microstructure. A convolution integral that characterizes the stress-strain time-dependent dependence is evaluated numerically. Numerical example to estimate the creep response of the structure for 2D and 3D application in a system made of concrete. The method can be used to a broad class of material with microstructure morphologies.

Various techniques and methods are applied for the study of creep phenomena, one of them being the dynamic relaxation (DR) technique, used in [20] to study the bending response of the Mindlin composite plate. A 3D micromechanical model is used for the mechanical identification of the unidirectional composite. The matrix is viscoelastic and nonlinear and is reinforced with elastic, unidirectional, isotropic transverse fibers. The viscoelastic constitutive equation Schapery [21] is used. Experimentally obtained results certify the correctness of the model used and the approach taken.

Creep response of structures manufactured by micro-heterogeneous composites can be approached as two-scale problem. It is well known that, at the microlevel, reinforced composites are characterized by a heterogeneous structure. This is determined by the response of the matrix, fibers and the bonding interphase. These non-homogeneities are neglected if a finite element analysis of such a material is made, considering that the properties of a single finite element are constant over the entire volume of the element. The paper aims to use a micromechanical model, based on obtaining the macro, average values, used for finite element modeling [22]. Thus, it results in the constitutive equations of the homogenized material, with values of engineering constants useful for practical applications. The values obtained are verified numerically.

The study the nonlinear creep behavior of different composite, considering the creep power law, using an empirical model, is proposed in [23,24]. This method is applied in [25,26] to a graphite/epoxy composites and measurements have validated the method. 

An analytical model that approximates the behavior of composites reinforced with short fibers, is proposed in [27]. A parametric study establishes the effects of geometric parameters on the creep deformation rate, which is the main objective of the work.

The equations that describe the proposed model satisfy the equations of equilibrium and constitutive creep equations. The model is then validated by the finite element method, observing a satisfactory concordance between the results obtained with the simplified model and the finite element method, more laborious and more expensive.

The Mori–Tanaka method, together with the additive interaction (AI) law for the calculation of the characteristics of viscoelastic materials, linear and nonlinear, are compared with:

(i) the results obtained in the literature using the (FEM) and the fast Fourier transform;
(ii) usual homogenization methods based on variational approaches;
(iii) analytical solutions exact which can be obtained by classical methods in linear viscoelasticity [28].

For the performed calculations are considered different configurations and geometries that may exist: spherical reinforcing materials, aligned unidirectional fibers, soft or hard materials, with significant difference between the phases involved. The method proves useful for approaching such a system, the calculations performed showing small differences between the classical results verified and tested experimentally and the proposed method.

In [29,30,31], a new method is used to study the time-dependent behavior of viscoelastic material by means of a variational principle. In this model, a composite material is modeled as a material reinforced with a collection of cylindrical fibers having different diameters. The average is made considering the strain energy of a representative unit element to be equal with the energy of the equivalent homogenized element.

There numerous papers in the domain, studying the overall properties of the bi and multiphasic materials [29,30,31,32]. New and interesting research in the field has deepened the results obtained previously and offered new development directions [33,34,35,36,37].

In the paper, the FEM is used to obtain the mechanical constants of a composite material reinforced with unidirectional fibers. The results are obtained for a composite reinforced with carbon fibers and experimental results are obtained in order to valid the calculus. A good agreement between the calculated values and the experimental results is observed. 

FEM is an advantageous way to approach this problem because, at the moment, it is a very well-developed method, with well-developed computer software, with an extremely rich experience in previous applications. The replacement of the laborious and expensive operations of experimental determination of the engineering constants of a material with the application of numerical procedures, well validated by practice, allows the facilitation of obtaining these values in a shorter time and with much reduced costs.

## 2. Constitutive Relations of Transversely Isotropic Media 

For a non-aging viscoelastic anisotropic material, under general loading states, the linear viscoelastic relation between stresses and strains can be expressed in a compact form using the Boltzmann’s superposition integral as:

(1) $$\varepsilon_{ij}(t)=\int_{-\infty }^{t}S_{ijkl}\left(t-\tau \right)\frac{\partial \sigma_{kl}\left(\tau \right)}{\partial \tau }d\tau ,\;$$

or in the inverse form as:

(2) $$\sigma_{ij}(t)=\int_{-\infty }^{t}C_{ijkl}\left(t-\tau \right)\frac{\partial \varepsilon_{kl}\left(\tau \right)}{\partial \tau }d\tau ,\;$$

It is worthwhile mentioning that the Boltzmann’s superposition principle is a consequence of linear material behavior and therefore may be applied only in the linear range. It should be pointed out that as a result of the symmetry of the strain and stress tensors, the creep compliances $S_{ijkl}\left(t\right)$ and the relaxation moduli $C_{ijkl}\left(t\right)$ tensors each containing 81 terms and, just as for elastic materials, are symmetric with respect to the interchange of index *i* with *j* and *k* with *l* [38]. This property has been verified analytically by Schapery [39] and was later experimentally proven by Morris et al. [40] to be true.

Consider, now, a generalized creep test in which, by definition,

(3) $$\sigma_{ij}(t)={\tilde{\sigma }}_{ij}H(t),\;$$

where all ${\tilde{\sigma }}_{ij}$ are constant. Substituting this relation into Equation (1) yields:

(4) $$\varepsilon_{ij}(t)=S_{ijkl}(t){\tilde{\sigma }}_{ij}.$$

Similarly, for a generalized stress relaxation test,

(5) $$\varepsilon_{ij}(t)={\tilde{\varepsilon }}_{ij}H(t)$$

where all ${\tilde{\varepsilon }}_{ij}$ are constant, it follows, from Equation (2) that:

(6) $$\sigma_{ij}(t)=C_{ijkl}\left(t\right){\tilde{\varepsilon }}_{kl}$$

Note that H(t) is the Heaviside function defined customarily as:

(7) $$H(t)=\left\{ \begin{array}{l} 0\;for\;t<0\\ 1\;for\;t\geq 0 \end{array}\right.\;,$$

with its derivative known as the Dirac delta function δ(t) having the following property:

(8) $$\delta (t)=\left\{ \begin{array}{l} 0\;for\;t\neq 0\\ \infty \;for\;t=0 \end{array}\right.\;.$$

**Figure 1:**
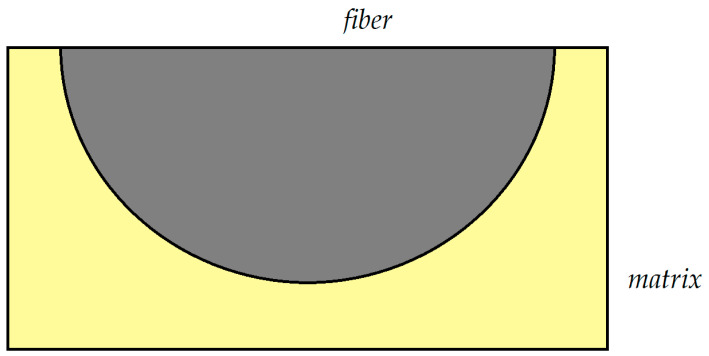
The representative unit cell for finite element analysis (Model 1).

If there is one plane in which the mechanical properties are the same in all directions, the material is termed “transversely isotropic”. Let us now consider a fiber-reinforced plastic in which all fibers are aligned in the “1” direction and are distributed randomly in the “2-3” plane. The specimen shown in Figure 1 fits this situation and can therefore be referred to as a transversely isotropic media with the “2-3” plane being the plane of isotropy. In this figure, the (x, y, z) and (1, 2, 3) axes are denoted as global and local coordinates, respectively.

Assuming isothermal conditions for a transversely isotropic material, under the generalized creep test, Equation (4) can be written in a reduced form as:

(9) $$\left\{\begin{array}{c} \varepsilon_{1}\\ \varepsilon_{2}\\ \varepsilon_{3}\\ \gamma_{23}\\ \gamma_{31}\\ \gamma_{12} \end{array}\right\}=\left[\begin{array}{cccccc} S_{11} & S_{12} & S_{12} & 0 & 0 & 0\\ S_{12} & S_{22} & S_{23} & 0 & 0 & 0\\ S_{12} & S_{12} & S_{22} & 0 & 0 & 0\\ 0 & 0 & 0 & S_{44} & 0 & 0\\ 0 & 0 & 0 & 0 & S_{66} & 0\\ 0 & 0 & 0 & 0 & 0 & S_{66} \end{array}\right]\left\{\begin{array}{c} \sigma_{11}\\ \sigma_{22}\\ \sigma_{33}\\ \tau_{23}\\ \tau_{31}\\ \tau_{12} \end{array}\right\}$$

where:

(10) $$S_{44}=2\left(S_{22}-S_{23}\right),$$

and:

(11) $$\gamma_{ij}=2\;\varepsilon_{ij}\;i,j\;=\;1,2,3\;\sigma_{ii}=\sigma_{i}\;,\;\varepsilon_{ii}=\varepsilon_{i}\;i\;=\;1,2,3\;(i\mathrm{not}\;\mathrm{summed}).$$

It is to be pointed out that $S_{23}=S_{32}$ because of the geometric symmetry present. In addition, all the strains and creep compliances in the above equations may in general be functions of time. Moreover, for the sake of notational simplicity the stresses have been written without the overbar.

For plane stress analysis, the above compliance matrix may depend on four independent functions of time.

(12) $$\left\{\begin{array}{c} \varepsilon_{1}\\ \varepsilon_{2}\\ \gamma_{12} \end{array}\right\}=\left[\begin{array}{ccc} S_{11} & S_{12} & 0\\ S_{12} & S_{22} & 0\\ 0 & 0 & S_{66} \end{array}\right]\left\{\begin{array}{c} \sigma_{1}\\ \sigma_{2}\\ \tau_{12} \end{array}\right\}$$

Furthermore, if the stresses acting on the composite are considered relative to the coordinates (1, 2, 3) oriented at an angle θ with the respect to the global coordinates (x, y, z), none of the above nine lamina compliances is zero. The strain–stress relation in the (x, y, z) coordinate system is therefore written as:

(13) $$\left\{\begin{array}{c} \varepsilon_{x}\\ \varepsilon_{y}\\ \gamma_{xy} \end{array}\right\}={\left[\begin{array}{ccc} S_{11} & S_{12} & S_{16}\\ S_{12} & S_{22} & S_{26}\\ S_{16} & S_{26} & S_{66} \end{array}\right]}_{G}\left\{\begin{array}{c} \sigma_{x}\\ \sigma_{y}\\ \tau_{xy} \end{array}\right\}$$

or:

(14) $${\left\{\varepsilon \right\}}_{xy}={\left[S\right]}_{G}{\left\{\sigma \right\}}_{xy}$$

The transformed compliance terms in ${\left[S\right]}_{G}$ are related to the basic lamina compliance terms, $S_{11}$, $S_{12}$, $S_{22}$ and $S_{66}$ by a rotational transformation which involves fourth power terms of sinθ and cosθ. 

Since the Laplace transform of the generalized Hooke’s Law: ${\hat{\varepsilon }}_{i}=S_{ij,G(p)}{\hat{\sigma }}_{j}$ represents the constitutive equation for a linear viscoelastic material [41], a time-dependent strain stress relation follows from Equation (13). Thus, using Equation (1), one can write:

(15) $$\varepsilon_{1}(t)=\int_{0}^{t}S_{11}\left(t-\tau \right)\frac{\partial \sigma_{1}\left(\tau \right)}{\partial \tau }d\tau +\int_{0}^{t}S_{12}\left(t-\tau \right)\frac{\partial \sigma_{2}\left(\tau \right)}{\partial \tau }d\tau$$

Here, $\varepsilon_{1}(t)$ is the strain in the direction “1” which coincides with the fiber direction. Similarly, the expression for $\varepsilon_{1}(t)$ oriented at an angle θ to the “1” axis is:

(16) $$\varepsilon_{x}(t)=\int_{0}^{t}S_{11,G}\left(t-\tau \right)\frac{\partial \sigma_{x}\left(\tau \right)}{\partial \tau }d\tau +\int_{0}^{t}S_{12,G}\left(t-\tau \right)\frac{\partial \sigma_{y}\left(\tau \right)}{\partial \tau }d\tau +\int_{0}^{t}S_{16,G}\left(t-\tau \right)\frac{\partial \tau_{xy}\left(\tau \right)}{\partial \tau }d\tau$$

where *t* is the whole time history of the composite and τ represent the time at which the stress σ(τ) is applied.

In a unidirectional composite, longitudinal deformation is mainly a fiber dominated property, while shear and transverse deformations are for the most part, matrix independent. In other words, in the compliance matrix, the components $S_{11}$ and $S_{12}$ are considered constants and may be determined from uniaxial tension tests performed on a 0° specimen. This is due to the fact that the fibers are much stiffer than the matrix; hence, the response of a lamina in the fiber direction is more or less controlled by the fiber properties. Thus, $S_{11}$—the compliance along the fiber direction—is taken as time-independent and $S_{12}=\nu_{12}S_{11}$ where $\nu_{12}$ is the major Poisson’s ratio. Griffith [42] showed that for T300/934 carbon/epoxy, the $S_{11}$ and $S_{12}$ components are fiber dominated compliances and are both time and stress independent band and therefore may be considered as linear elastic material properties. Even with the assumption of elastic fibers, in a composite material the creep compliances $S_{11}$ and $S_{12}$ may show slight time independence. This observation is due to the relaxation of the matrix in a fixed grip configuration and is not caused by creep of either component [41]. Clearly, in a creep test, both constituents of a composite, namely fiber and resin, experience nearly the same strain. Therefore, as the material extends under the applied load, a certain amount of the stress is imposed onto fiber and matrix. Since the fibers are elastic and hence do no creep, the viscoelastic matrix being now restrained from creep undergoes stress relaxation. As a consequence of this, part of the load is transferred to the fibers resulting in a small secondary strain, which is normally taken as creep. This phenomenon is referred to as “relaxation-creep” and is essentially dependent on the fiber response.

The component $S_{22}$ is on the other hand, the compliance transverse to the fiber direction and is determined from creep tests on the 90° specimen. This property is on the other hand, matrix dominated since it is closely related to the matrix response.

The remaining $S_{66}$ compliance may be obtained from uniaxial tension test on the 10° of axis specimen. The compliance of 10° specimen, is first determined and afterwards $S_{66}$ is obtained by using the transformation relationship [43]. In other words, the axial compliance $D_{\theta }\equiv \varepsilon_{x}/\sigma_{x}$ can be determined from an axially mounted strain gage on an off-axis tensile specimen. By expanding the first term of Equation (13), the axial compliance can be related to the principal compliances in Equation (12). It follows that:

(17) $$S_{11,G}\equiv D_{\theta }=S_{xx}=S_{11}\cos^{4}\theta +S_{22}\sin^{4}\theta +\left(2S_{12}+S_{66}\right)\sin^{2}\theta \cos^{2}\theta$$

Knowing $D_{\theta }$, the shear compliance $S_{66}$ can be solved for, in terms of the known values of $S_{11},S_{22},S_{12}$ and the angle θ. Measurement of $S_{66}$ is of great importance to the designer since it provides information about the time dependent shear behavior of the composite body. Moreover, the matrix plays a dominant role in the transfer of stress, thus requiring careful measurement of the intra-laminar or inter-laminar shear behavior.

In addition, the off-axis tensile test mentioned above, there are several other mechanical tests which are considered as suitable candidates to provide information about shear behavior of composite; torsion test, short beam bending tests and tensile test on ${\left[\pm 45\right]}_{s}$ coupons. The latter appears to be one of the most suitable tests for the viscoelastic measurements particularly those of the time dependent shear modulus $G_{12}$. This laminate configuration has been used in the present investigation to measure shear behavior.

## 3. Finite Element Procedures

**Figure 2:**
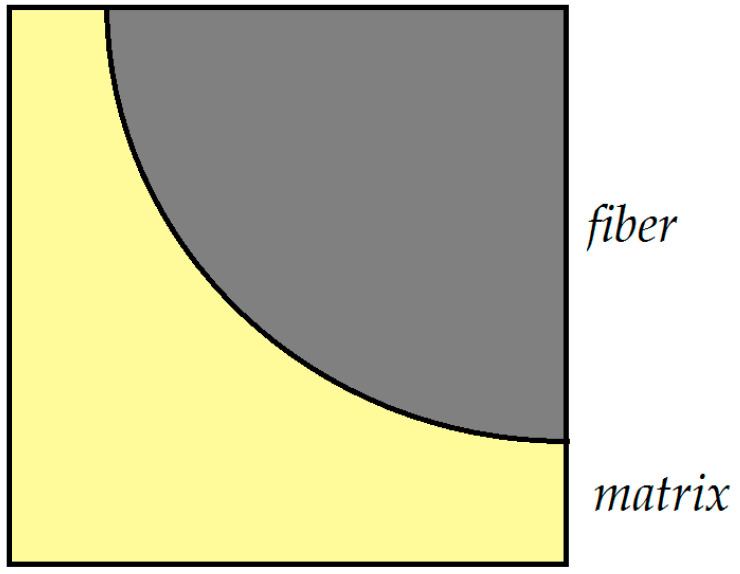
The representative unit cell for finite element analysis (Model 2).

The FEM is a powerful tool to study the response of composite materials [44]. For instance, the influence of temperature variation and dilatations was investigated in a unidirectional graphite/epoxy using a finite element numerical analysis by Adams et al [45]. In another investigation made by Wisnom [46], a unidirectional continuous silicon carbide fiber was studied. Here, like in the present study, a section of one quarter of a fiber was modeled using a nonlinear finite element analysis program. Brinson et al. [47] used a similar model as just described to study the global composite moduli. In the paper a finite element analysis is used to evaluate the fields of stresses and strains [48] in a transversely isotropic composite. By use of the FEM, the internal stresses as well as strains in the material are examined by constructing a finite element mesh of the internal structure of a unidirectional composite under various external loading conditions. For this purpose, the repeating unit cell can be taken as the finite element model. However, the symmetry of the model allows the analysis to be performed on only a quarter or half of the unit cell, the so called “representative unit cell” (RUC). This type of symmetry will reduce the difficulty of the analysis. The finite element unit cell model and the coordinate system used in the present study in shown in the Figure 1 and Figure 2 (Model 1 and 2). Quadrilateral elements with 8 nodes for plane-strain analysis were used for the finite element mesh of the model. Three variants were considered for this model: one with 9000 elements (Model 2-a) and the other with 18,000 elements (Model 2-b). The results obtained from both of these variants were practically the same. The number of elements was increased to 25,000 in a third variant (Model 2-c).

The same type of variation with respect to the number of elements and applied loads was also exercised with the representative model consisting of half the fiber with the corresponding matrix material (Model 1) for which the model is shown in Figure 1. Here, the variant with 16,000 elements (Model 1-a) supplied the same results as the one with 36,000 elements (Model 1-b). 

In order to demonstrate the potential application of the analysis, the above micromechanical/finite element model is applied to the glass/epoxy composite [49]. The time independent material properties for the constituents of the above composite are given below:

*E_m_ =* 4.14 GPa; *v_m_ =* 0.22; *E_f_ =* 86.90 GPa; *v_f_* = 0.34

It should be pointed out that the fiber volume percent used for the computation of elastic characteristic parameters as given in [50] is $v_{f}=63\%$. However, for the composites of the present investigation $v_{f}=60\%$ was used in all calculations.

Note that in Figure 2 all displacements in x2-direction along the right-hand boundary corresponding to $x_{2}=R+h/2$ are considered to be equal. In addition, all displacements in the $x_{3}$ direction, considered along $x_{3}=\pm \left(R+h/2\right)$ are equal in magnitude. Furthermore, both of the above boundaries can move freely in the $x_{3}$ and $x_{2}$ directions, respectively. The boundaries $x_{2}=0$ and $x_{3}=0$ are taken to be fixed in the $x_{2}$ and $x_{3}$ directions respectively, while being free to move in the $x_{2}$ and $x_{3}$ directions, respectively. In addition, all out-of-plane normal strains in the $x_{1}$ direction are considered to be equal in magnitude, which implies equal displacements in the $x_{1}$ direction. Similar boundary conditions like those mentioned above can be applied as well to the 3-D model.

In the present analysis the nodal point values of stress and strain are provided by the finite element program. These are used to compute the average values of the stresses and strains in each of the constituents and in the representative unit cell. With the above values, one can determine the elastic moduli and Poisson’s ratios according to the relations which follow. The results of the analysis are shown in Table 1, Table 2, Table 3, Table 4, Table 5 and Table 6.

It should be mentioned that the remaining loading conditions (case 4 and 5) were also exercised with the indicated models for which analogous results were obtained but for the sake of brevity are not presented here.

Comparison of the results of the foregoing models reveals that the different loading conditions provide the same results for the elastic moduli as well as for other characteristic parameters.

Finally, a three-dimensional model was built in order to calculate shear modulus and Poisson’s ratios in a plane perpendicular to $x_{2}x_{3}$.

A few of the foregoing models are listed in Table 7 for which the results using finite element analysis are obtained. For these models, the average stresses and the average strains in the subcell as well as those in the (RUC) are computed. These values are then used to evaluate the elastic constants such as shear moduli.

They can exist some discrepancy between the present FE results and those presented in [49]. With respect to these discrepancies, the following verification should be considered.

If the boundary condition for FE model is taken as $u_{i}=\alpha_{ij}x_{j}$ (where $\alpha_{ij}=\alpha_{ji}$), the average strain should be equal to ${\bar{\varepsilon }}_{ij}=\alpha_{ij}$**.** This can be demonstrated as follows:

(18) $${\bar{\varepsilon }}_{ij}=\frac{1}{V}\int_{\Gamma }\varepsilon_{ij}dV=\frac{1}{2V}\int_{\Gamma }\left(\frac{\partial u_{j}}{\partial x_{i}}+\frac{\partial u_{i}}{\partial x_{j}}\right)dV.$$

By applying Green’s theorem, it follows that:

(19) $${\bar{\varepsilon }}_{ij}=\frac{1}{2V}\int_{\partial \;\Gamma }\left(n_{i}u_{j}+n_{j}u_{i}\right)ds=\;=\frac{1}{2V}\left(\int_{\partial \;\Gamma }n_{i}\alpha_{jk}x_{k}ds+\int_{\partial \;\Gamma }n_{j}\alpha_{il}x_{l}ds\right)=\;=\frac{1}{2V}\left(\alpha_{jk}\int_{\partial \;\Gamma }n_{i}x_{k}ds+\alpha_{il}\int_{\partial \;\Gamma }n_{j}x_{l}ds\right).$$

Using Green’s theorem, the above equation can be written as follows:

(20) $${\bar{\varepsilon }}_{ij}=\frac{1}{2V}\left(\alpha_{jk}\int_{\;\Gamma }\frac{\partial x_{k}}{\partial x_{i}}dV+\alpha_{il}\int_{\;\Gamma }\frac{\partial x_{l}}{\partial x_{j}}dV\right)=\;=\frac{1}{2V}\left(\alpha_{jk}\int_{\;\Gamma }\delta_{ki}dV+\alpha_{il}\int_{\;\Gamma }\delta_{lj}dV\right)=\frac{1}{2V}\left(\alpha_{ji}+\alpha_{ij}\right)=\alpha_{ij}$$

In the present study the boundary conditions, $u_{i}=\alpha_{ii}x_{i}$ was used. It is therefore expected that ${\bar{\varepsilon }}_{ii}=\alpha_{ii}$ and ${\bar{\varepsilon }}_{ij}=0$ for i≠j. The results obtained are completely in accordance with the theory. The discrepancy between the current results and those presented in [49] may be due to the different type of finite elements used.

As mentioned earlier, the method of finite elements is used to obtain average values of strains and stresses, viz. ${\bar{\sigma }}_{22},{\bar{\sigma }}_{33},{\bar{\sigma }}_{11},{\bar{\sigma }}_{23}={\bar{\tau }}_{23},{\bar{\varepsilon }}_{22},{\bar{\varepsilon }}_{33},{\bar{\varepsilon }}_{11},{\bar{\varepsilon }}_{23}=1/2\;\gamma_{23}$ for the condition of plane strain which was considered for the present problem.

Next, the material constants of the unidirectional composite are evaluated [51]. The rule of mixture is applied to compute the longitudinal elastic modulus $E_{11}$ as follows:

(21) $$E_{11}=E_{f}\nu_{f}+E_{m}\nu_{m}$$

where $A=A_{f}+A_{m}$ and:

(22) $$\nu_{f}=\frac{A_{f}}{A}\;;\;\nu_{f}=\frac{A_{m}}{A}\;,$$

with $A_{f}$ is the cross section of the fiber and $A_{m}$ the cross section of the matrix.

In the plane strain case, the following relation can be written:

(23) $${\bar{\sigma }}_{22}=C_{22}{\bar{\varepsilon }}_{22}+C_{23}{\bar{\varepsilon }}_{33};\;{\bar{\sigma }}_{33}=C_{23}{\bar{\varepsilon }}_{22}+C_{22}{\bar{\varepsilon }}_{33};\;{\bar{\sigma }}_{11}=C_{12}\left({\bar{\varepsilon }}_{22}+{\bar{\varepsilon }}_{33}\right);\;{\bar{\tau }}_{23}=C_{66}\;{\bar{\gamma }}_{23},$$

from where it results:

(24) $$\left[\begin{array}{cc} {\bar{\varepsilon }}_{22} & {\bar{\varepsilon }}_{33}\\ {\bar{\varepsilon }}_{33} & {\bar{\varepsilon }}_{22} \end{array}\right]\left\{\begin{array}{c} C_{22}\\ C_{23} \end{array}\right\}=\left\{\begin{array}{c} {\bar{\sigma }}_{22}\\ {\bar{\sigma }}_{33} \end{array}\right\}$$

from where it results:

(25) $$\left\{\begin{array}{c} C_{22}\\ C_{23} \end{array}\right\}=\frac{1}{{\bar{\varepsilon }}_{22}^{2}-{\bar{\varepsilon }}_{33}^{2}}\left[\begin{array}{cc} {\bar{\varepsilon }}_{22} & -{\bar{\varepsilon }}_{33}\\ -{\bar{\varepsilon }}_{33} & {\bar{\varepsilon }}_{22} \end{array}\right]\left\{\begin{array}{c} {\bar{\sigma }}_{22}\\ {\bar{\sigma }}_{33} \end{array}\right\}$$

It is now possible to obtain the expression for $C_{22}$ and $C_{23}$:

(26) $$C_{22}=\frac{{\bar{\sigma }}_{22}{\bar{\varepsilon }}_{22}-{\bar{\sigma }}_{33}{\bar{\varepsilon }}_{33}}{{\bar{\varepsilon }}_{22}^{2}-{\bar{\varepsilon }}_{33}^{2}}\;;\;C_{23}=\frac{{\bar{\sigma }}_{33}{\bar{\varepsilon }}_{22}-{\bar{\sigma }}_{22}{\bar{\varepsilon }}_{33}}{{\bar{\varepsilon }}_{22}^{2}-{\bar{\varepsilon }}_{33}^{2}}\;$$

For $C_{12}$ and $C_{66}$:

(27) $$C_{12}=\frac{{\bar{\sigma }}_{11}}{{\bar{\varepsilon }}_{22}+{\bar{\varepsilon }}_{33}}\;;\;C_{66}=\frac{{\bar{\tau }}_{23}}{{\bar{\gamma }}_{23}}\;$$

Note that in the present study, the fibers are oriented along the “1” direction and distributed randomly in the “2-3” plane which is referred to as the plane of isotropy. For this transversely isotropic body, one can utilize the above constants to compute the bulk modulus $K_{23}$ in the plane “2-3” as shown below:

(28) $$K_{23}=\frac{C_{22}+C_{33}}{2}=\frac{{\bar{\sigma }}_{22}+{\bar{\sigma }}_{33}}{2\left({\bar{\varepsilon }}_{22}+{\bar{\varepsilon }}_{33}\right)}\;.$$

For the longitudinal Poisson’s ratio, one obtains:

(29) $$\nu_{1}=\nu_{21}=\nu_{31}=\frac{1}{2}{\left(\frac{C_{11}-E_{11}}{K_{23}}\right)}^{1/2}=\frac{C_{12}}{C_{22}+C_{33}}=\frac{{\bar{\sigma }}_{11}}{\left({\bar{\sigma }}_{22}+{\bar{\sigma }}_{33}\right)}\;.$$

The shear modulus can be either determined from:

(30) $$G_{23}=\frac{C_{22}-C_{33}}{2}=\frac{{\bar{\sigma }}_{22}-{\bar{\sigma }}_{33}}{2\left({\bar{\varepsilon }}_{22}-{\bar{\varepsilon }}_{33}\right)}\;.$$

or from:

(31) $$G_{23}=C_{66}=\frac{{\bar{\sigma }}_{23}}{2{\bar{\varepsilon }}_{23}}\;.$$

By introducing the following parameter,

(32) $$\psi =1+\frac{4\nu_{1}^{2}K_{23}}{E_{11}}\;,$$

the transverse moduli and Poisson’s ratio can then be expressed as:

(33) $$E_{22}=E_{33}=\frac{4G_{23}K_{23}}{K_{23}+\psi G_{23}}\;,$$

and:

(34) $$\nu_{23}=\frac{K_{23}-\psi G_{23}}{K_{23}+\psi G_{23}}\;,$$

respectively.

Up to now, the expressions for $E_{11},\;E_{22}=E_{33},\;\nu_{12}=\nu_{13},\;\nu_{23},\;G_{23},K_{23}$ were determined. A few observations should be made at this point regarding the above expressions. 

From the following relations:

(35) $$C_{22}+C_{33}=2K_{23}\;;\;C_{22}-C_{33}=2G_{23}\;,$$

one may obtain:

(36) $$C_{22}=K_{23}+G_{23}\;;\;C_{23}=K_{23}-G_{23}\;.$$

Recall that

(37) $$C_{44}=G_{1}=G_{12}=G_{13}\;;\;C_{12}=\nu_{1}\left(C_{22}+C_{23}\right)=2\nu_{1}K_{23}\;$$

and:

(38) $$C_{11}=E_{11}+\frac{2C_{12}^{2}}{C_{22}+C_{23}}=E_{11}+4\nu_{1}^{2}K_{23}=\psi E_{11}\;.$$

If the values for the material constants are known, one can calculate the above coefficients using the equations presented.

In a next step the FEM was used to compute the average stresses and strains in a three-dimensional elastic body. These are: ${\bar{\sigma }}_{11},{\bar{\sigma }}_{22},{\bar{\sigma }}_{33},{\bar{\sigma }}_{12}={\bar{\tau }}_{12},{\bar{\sigma }}_{23}={\bar{\tau }}_{23},{\bar{\sigma }}_{31}={\bar{\tau }}_{31}$, ${\bar{\varepsilon }}_{11},{\bar{\varepsilon }}_{22},{\bar{\varepsilon }}_{33},{\bar{\varepsilon }}_{12}=1/2\;\gamma_{12},{\bar{\varepsilon }}_{23}=1/2\;\gamma_{23},{\bar{\varepsilon }}_{31}=1/2\;\gamma_{31}$. Using the above components of stress and strain, the elastic constants which define a transversely isotropic composite, can be determined. The following relations can be written from the general Hooke’s Law:

(39) $${\bar{\sigma }}_{11}=C_{11}{\bar{\varepsilon }}_{11}+C_{12}{\bar{\varepsilon }}_{22}+C_{12}{\bar{\varepsilon }}_{33}\;{\bar{\sigma }}_{22}=C_{12}{\bar{\varepsilon }}_{11}+C_{22}{\bar{\varepsilon }}_{22}+C_{23}{\bar{\varepsilon }}_{33}\;{\bar{\sigma }}_{33}=C_{12}{\bar{\varepsilon }}_{11}+C_{23}{\bar{\varepsilon }}_{22}+C_{22}{\bar{\varepsilon }}_{33}\;{\bar{\sigma }}_{23}={\bar{\tau }}_{23}=\left(C_{11}-C_{23}\right){\bar{\varepsilon }}_{23}=\frac{1}{2}\left(C_{11}-C_{23}\right){\bar{\gamma }}_{23}\;{\bar{\sigma }}_{31}={\bar{\tau }}_{31}=2C_{44}{\bar{\varepsilon }}_{31}=C_{66}{\bar{\gamma }}_{31}\;{\bar{\sigma }}_{12}={\bar{\tau }}_{12}=2C_{44}{\bar{\varepsilon }}_{12}=C_{66}{\bar{\gamma }}_{12}$$

From the last of Equation (39) it is readily seen that

(40) $$C_{44}=\frac{{\bar{\sigma }}_{12}}{2\;{\bar{\varepsilon }}_{12}}=\frac{{\bar{\tau }}_{12}}{{\bar{\gamma }}_{12}}=G_{12}=G_{13}=G_{1}$$

which is the shear modulus in a plane normal to *x_2_x_3_* plane. Subtraction of the third equation from the second in Equation (39) yields

(41) $${\bar{\sigma }}_{22}-{\bar{\sigma }}_{33}=\left(C_{22}-C_{33}\right)\left({\bar{\varepsilon }}_{22}-{\bar{\varepsilon }}_{33}\right)$$

which can replace the fourth relation in Equation (39). This means that there exists a system of six equations, from which four are independent and they contain five unknowns. Thus, only four of the elastic constants can be solved for. One can, however, make use of the law of mixture, written here again for convenience, to compute the longitudinal Young’s modulus $E_{11}$:

(42) $$E_{11}=E_{f}v_{f}+E_{m}v_{m}$$

With the above expression for $E_{11}$ one can replace the redundant fourth relation with:

(43) $$E_{11}=C_{11}-\frac{2C_{12}^{2}}{C_{22}+C_{23}}.$$

Addition of the second and third equation in Equation (39) yields:

(44) $${\bar{\sigma }}_{22}+{\bar{\sigma }}_{33}-\frac{2C_{12}{\bar{\varepsilon }}_{11}}{{\bar{\varepsilon }}_{22}+{\bar{\varepsilon }}_{33}}=C_{22}+C_{23}.$$

From the first of Equation (39) one can show that:

(45) $$C_{11}=\frac{{\bar{\sigma }}_{11}-C_{12}\left({\bar{\varepsilon }}_{22}+{\bar{\varepsilon }}_{33}\right)}{{\bar{\varepsilon }}_{11}}.$$

Substitution of this relation into that of $E_{11}$ yields:

(46) $$E_{11}=\frac{{\bar{\sigma }}_{11}}{{\bar{\varepsilon }}_{11}}+C_{12}\frac{{\bar{\varepsilon }}_{22}+{\bar{\varepsilon }}_{33}}{{\bar{\varepsilon }}_{11}}-\frac{2C_{12}^{2}\left({\bar{\varepsilon }}_{22}+{\bar{\varepsilon }}_{33}\right)}{\left({\bar{\sigma }}_{22}+{\bar{\sigma }}_{33}-2C_{12}{\bar{\varepsilon }}_{11}\right)}.$$

and this equation enables one to compute the coefficient $C_{12}$. Other methods to compute these coefficients can be found in [52,53].

## 4. Experimental Creep Response of Fiber Reinforced Composite

**Figure 3:**
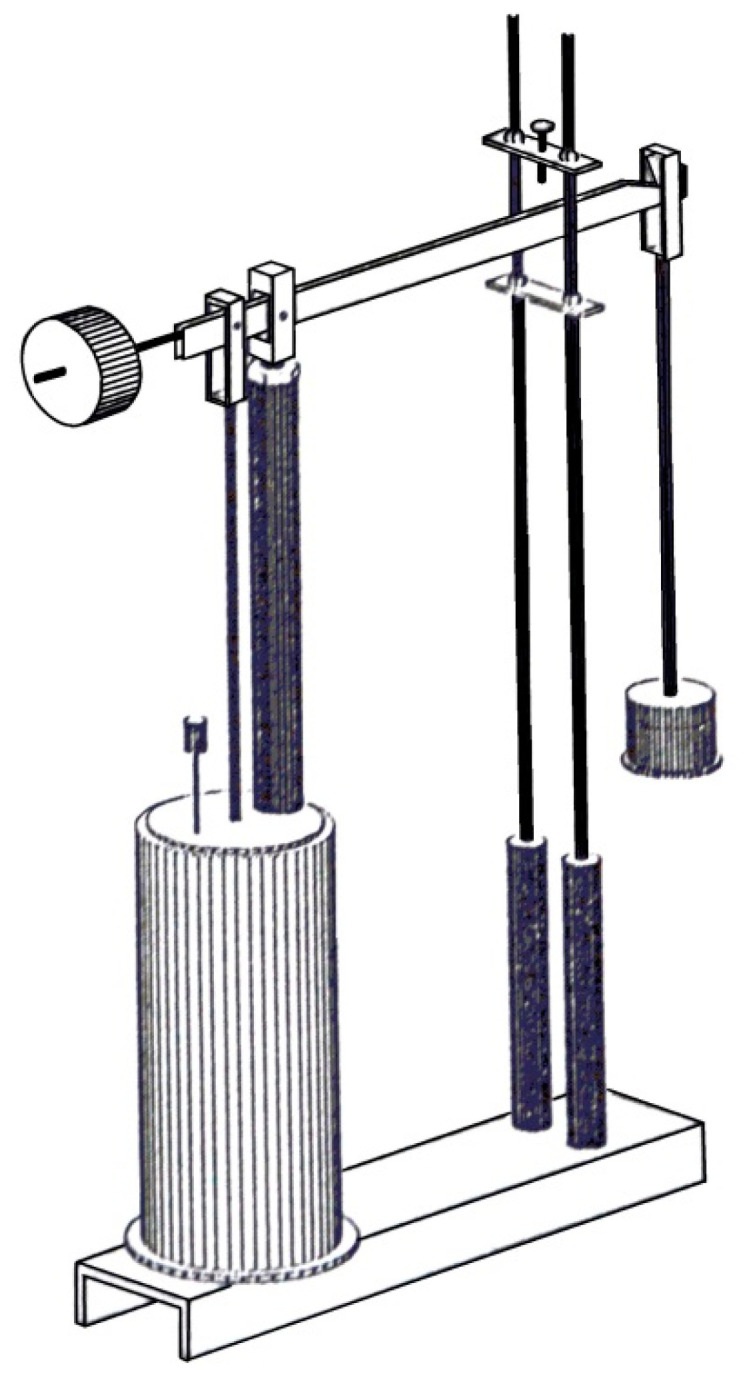
Experimental testing device. Single lever device.

**Figure 4:**
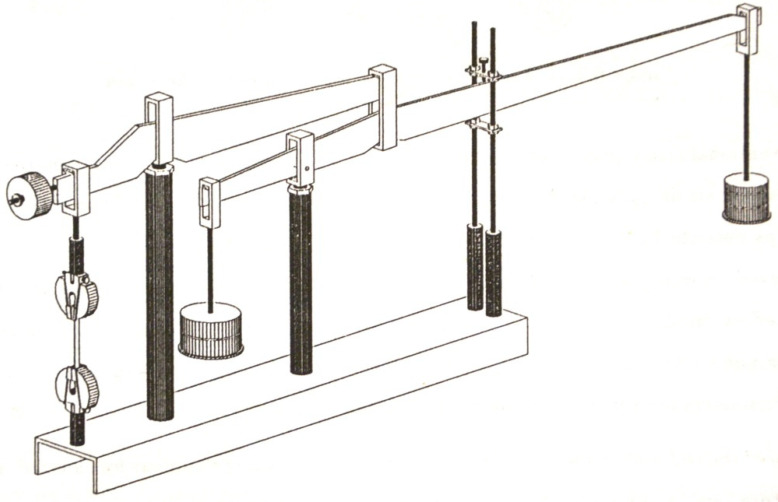
Experimental testing device. Double lever device.

Once the mechanical constants are computed, using the above obtained formulas, it is possible to have a theoretical creep response of the materials. An experimental procedure offers us a verification of the computed results. In Figure 3 and Figure 4, the experimental testing device are presented in two variants: with one single lever and with two levers.

**Figure 5:**
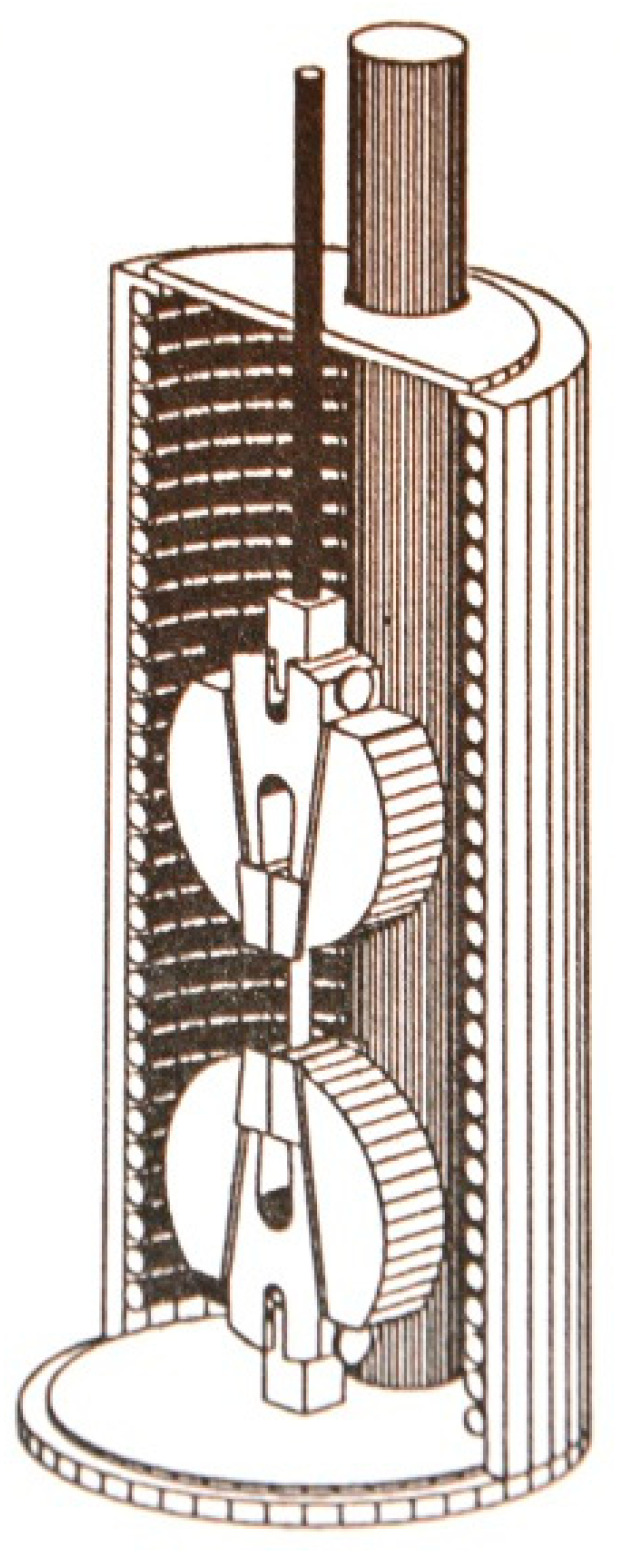
The cylindrical heating chamber for elevated temperature testing.

The experiments will be performed in order to determine the behavior of carbon fiber composite. The test specimens are subjected to different stress and different temperatures. A cylindrical heating chamber for elevated temperatures is presented in Figure 5. The relative humidity in this chamber was 35%. 

The test program comprises isothermal testing at room and elevated temperatures. Room temperature was 23 °C and ambient humidity. The program is to test the specimen over a period of 10 h. Single and double lever arrangement have ratios of 10:1 and 25:1 respectively.

The test specimens have the following dimensions: length of 150 mm, width of 10 mm and thickness of 1 mm. These dimensions are valuable for all the test specimens. Before performing tests, the test specimens have been stored in desiccants filled chamber (to protect from humidity: the relative humidity in this chamber was 35%).

The experiments were made on commercially available composites. The material used is an epoxy Fibredux 6376C, reinforced with carbon fibers T800. Another thermoplastic material was APC2, reinforced with carbon fibers IM6. 

**Figure 6:**
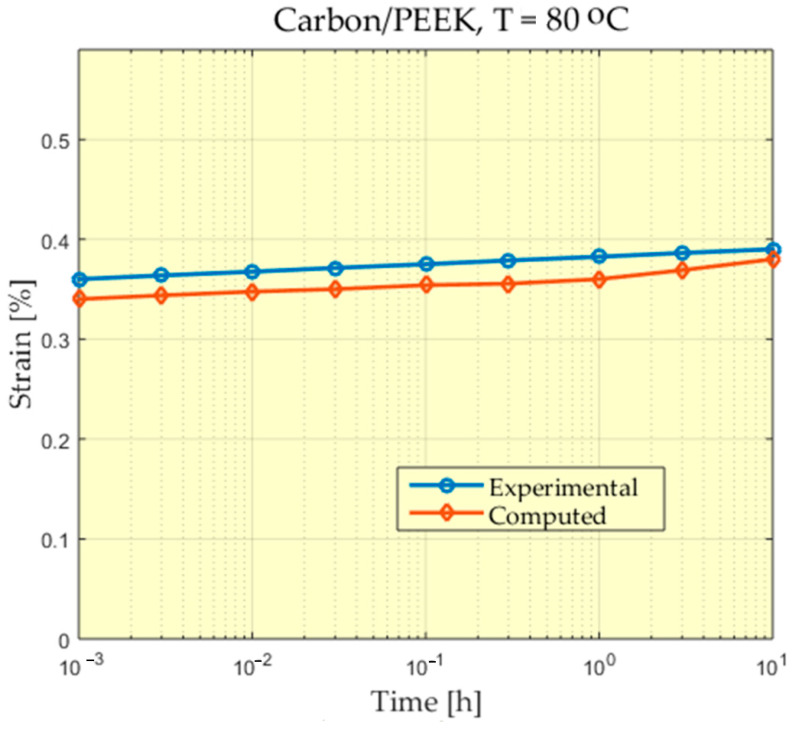
Comparison between experimental and theoretical prediction of creep strain for Carbon/PEEK subjected to 26 MPa at 80 °C.

**Figure 7:**
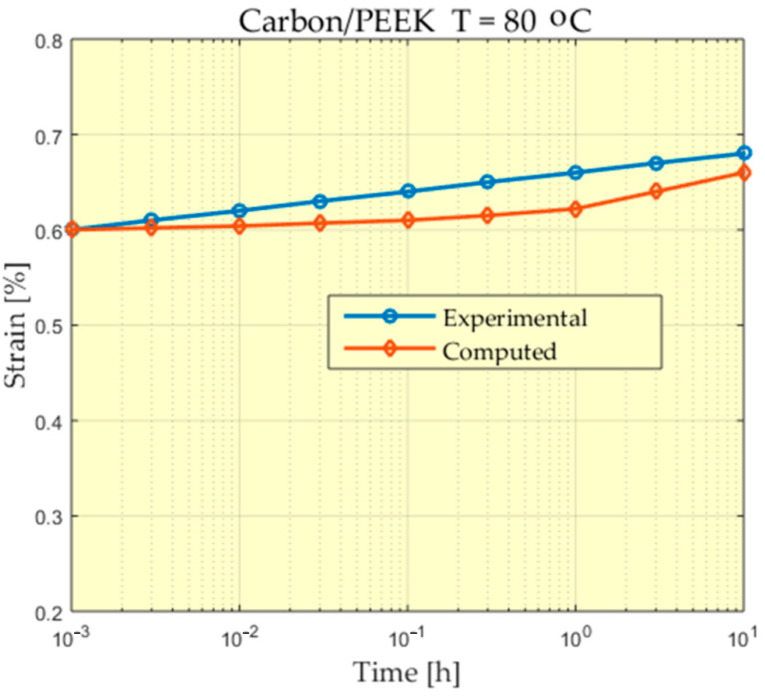
Comparison between experimental and theoretical prediction of creep strain for Carbon/PEEK subjected to 47 MPa at 80 °C.

**Figure 8:**
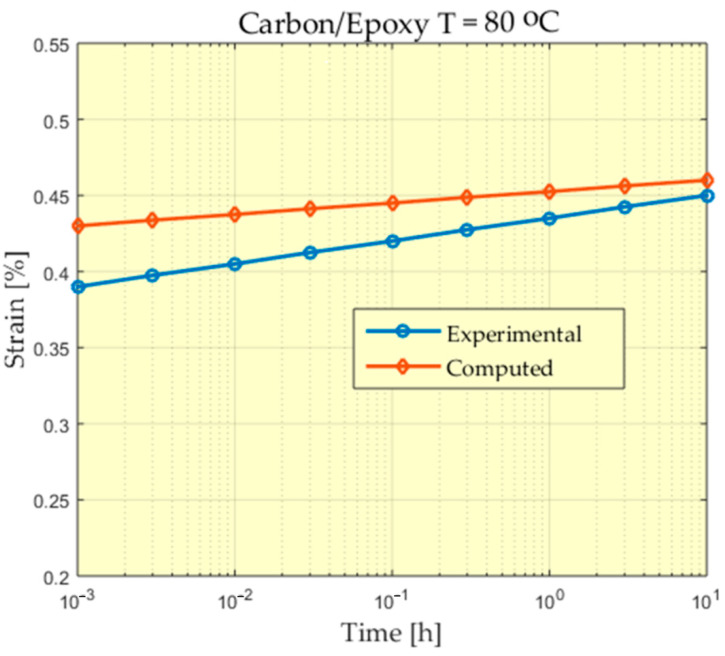
Comparison between experimental and theoretical prediction of creep strain for Carbon/Epoxy subjected to 30 MPa at 80 °C.

**Figure 9:**
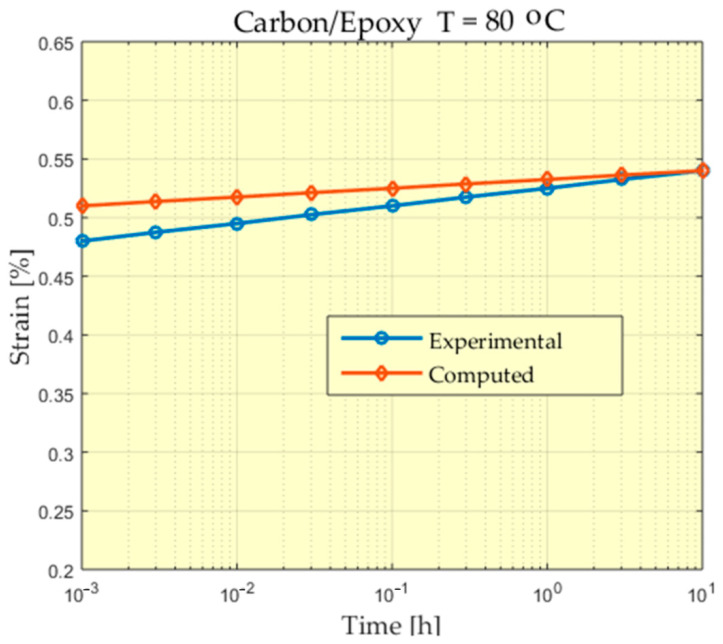
Comparison between experimental and theoretical prediction of creep strain for Carbon/Epoxy subjected to 36 MPa at 80 °C.

The experimental behavior of a Carbon/PEEK material at the temperature of 80 °C is presented in Figure 6, Figure 7, Figure 8 and Figure 9. 

For some of the experiments a greater difference can be observed between the measured values and the values calculated at the beginning of the experiment. A study of these differences could be made, taking into account that the scale used for time is a logarithmic scale and the time in which these differences appear is very short compared to the total time of the experiment. In addition, these differences appear at the beginning of the experiment when, from the value of zero, these deformations increase, reaching values that begin to stabilize. 

## 5. Conclusions and Discussion

The finite element method can be an appropriate calculation method to determine the overall properties of multi-phase composite materials. The averaging of the written equations presents the possibility to estimate the engineering constants, necessary for the performed calculations. To verify the results obtained, experimental tests were performed using Carbon/Epoxy and Carbon/PEEK. The tests described in the paper show a good concordance between the obtained results and the experimental verifications. In this way, the finite element method proves to be a powerful tool for calculating the mechanical properties of a multiphase material. Compared to the already known and applied methods, such as the use of micromechanical models, homogenization theory and Mori–Tanaka formalism, the finite element method is added, as a useful and relatively simple method of determining the coefficients of the constitutive equations of the material [54]. A future way of developing the topic is to improve the procedure by adding new results on how the finite element method can be used together with a parametric approach to the problem.

In the paper, several aspects of the behavior of a material in the case of flow tests were studied. For creep phenomena, the influences of temperature prove to be nonlinear. Unidirectional composite materials with transverse isotropic behavior, but having an elastic behavior, are studied. The composite material obtained has a viscoelastic behavior.

The presented method can replace the experimental determination of the mechanical properties of a viscoelastic material with calculations made by applying the finite element method on simple mechanical models.

## Figures and Tables

**Table 1:** Average values of stress and strain (Case 1).

$\bar{\sigma }$ ^a^	Fiber	Matrix	RUC ^b^	$\bar{\varepsilon }$ ^c^	Fiber	Matrix	RUC
${\bar{\sigma }}_{22}$	0.146 × 10^3^	0.122 × 10^3^	0.138 × 10^3^	${\bar{\varepsilon }}_{22}$	0.16 × 10^0^	0.263 × 10^1^	0.106 × 10^1^
${\bar{\sigma }}_{33}$	0.71 × 10^0^	−0.906 × 10^0^	0.118 × 10^0^	${\bar{\varepsilon }}_{33}$	−0.445 × 10^−1^	−0.137 × 10^1^	−0.529 × 10^0^
${\bar{\sigma }}_{11}$	0.324 × 10^2^	0.413 × 10^3^	0.357 × 10^2^	${\bar{\varepsilon }}_{11}$	0.0 × 10^0^	0.0 × 10^0^	0.0 × 10^0^
${\bar{\sigma }}_{23}$	0.194 × 10^−4^	0.412 × 10^2^	0.359 × 10^−4^	${\bar{\varepsilon }}_{23}$	0.539 × 10^−7^	0.447 × 10^−5^	0.167 × 10^−5^

^a^ Average stress; ^b^ representative unit cell; ^c^ average strain.

**Table 2:** Computed values of elastic moduli (Case 1).

Modulus [MPa]	Matrix	Fiber	Average
$E_{11}$	4140.0	86,900.0	56,278.0
$E_{23}=E_{13}$	4140.0	86,899.0	12,741.0
$\nu_{1}$	0.34	0.22	0.259
$\nu_{23}$	0.34	0.22	0.475
$G_{23}$	1544.0	35,614.7	4318.2
$K_{23}$	4827.4	63,597.7	12,886.2

**Table 3:** Average values of stress and strain (Case 2).

$\bar{\sigma }$	Fiber	Matrix	RUC	$\bar{\varepsilon }$	Fiber	Matrix	RUC
${\bar{\sigma }}_{22}$	0.147 × 10^3^	0.122 × 10^3^	0.138 × 10^3^	${\bar{\varepsilon }}_{22}$	0.134 × 10^0^	0.183 × 10^1^	0.757 × 10^0^
${\bar{\sigma }}_{33}$	0.857 × 10^2^	0.702 × 10^2^	0.801 × 10^2^	${\bar{\varepsilon }}_{33}$	0.485 × 10^−1^	0.157 × 10^0^	0.881 × 10^−1^
${\bar{\sigma }}_{11}$	0.512 × 10^2^	0.653 × 10^2^	0.564 × 10^2^	${\bar{\varepsilon }}_{11}$	0.0 × 10^0^	0.0 × 10^0^	0.0 × 10^0^
${\bar{\sigma }}_{23}$	0.992 × 10^−5^	0.322 × 10^−4^	0.181 × 10^−4^	${\bar{\varepsilon }}_{23}$	0.306 × 10^−7^	0.190 × 10^−5^	0.717 × 10^−6^

**Table 4:** Computed values of elastic moduli (Case 2).

Modulus	Matrix	Fiber	Average
$E_{11}$	4140.0	86,900.0	56,278.0
$E_{23}=E_{13}$	4140.0	86,900.0	12,741.0
$\nu_{1}$	0.34	0.22	0.259
$\nu_{23}$	0.34	0.22	0.475
$G_{23}$	1544.0	35,614.8	4318.2
$K_{23}$	4827.4	63,597.8	12,886.2

**Table 5:** Average values of stress and strain (Case 3).

$\bar{\sigma }$	Fiber	Matrix	RUC	$\bar{\varepsilon }$	Fiber	Matrix	RUC
${\bar{\sigma }}_{22}$	0.147 × 10^3^	0.123 × 10^3^	0.138 × 10^3^	${\bar{\varepsilon }}_{22}$	0.160 × 10^0^	0.263 × 10^1^	0.106 × 10^−1^
${\bar{\sigma }}_{33}$	0.644 × 10^0^	−0.983 × 10^0^	0.049 × 10^−1^	${\bar{\varepsilon }}_{33}$	−0.446 × 10^−1^	−0.137 × 10^1^	0.530 × 10^0^
${\bar{\sigma }}_{11}$	0.324 × 10^2^	0.414 × 10^2^	0.357 × 10^2^	${\bar{\varepsilon }}_{11}$	0.0 × 10^0^	0.0 × 10^0^	0.0 × 10^0^
${\bar{\sigma }}_{23}$	0.899 × 10^−5^	−0.979 × 10^−4^	−0.301 × 10^−4^	${\bar{\varepsilon }}_{23}$	0.258 × 10^−7^	−0.638 × 10^−5^	0.232 × 10^−5^

**Table 6:** Computed values of elastic moduli (Case 3).

Modulus	Matrix	Fiber	Average
$E_{11}$	4140.0	86,900.0	56,279.0
$E_{23}=E_{13}$	4140.0	86,899.0	12,754.0
$\nu_{1}$	0.34	0.22	0.259
$\nu_{23}$	0.34	0.22	0.475
$G_{23}$	1544.0	35,614.7	4322.2
$K_{23}$	4827.4	63,597.7	12,900.8

**Table 7:** Description of finite element models and associated boundary conditions (BC).

Case	Model	BC (x_2_ Direction)	BC (x_3_ Direction)
1	Model 1-a	*p_x_* = 137.90 (MPa)	*p_y_* = 0.00 (MPa)
2	Model 1-b	*p_x_* = 137.90 (MPa)	*p_y_* = 80.0 (MPa)
3	Model 2-a	*p_x_* = 137.90 (MPa)	*p_y_* = 0.00 (MPa)
4	Model 2-b	*u_x_* = 0.01 (mm)	*u_y_* = 0.01 (mm)
5	Model 2-c	*u_x_* = 0.01 (mm)	*u_y_* = 0.01 (mm)

## Data Availability

Not applicable.
